# Retro Mode Imaging for Detection and Quantification of Sub-RPE Drusen and Subretinal Drusenoid Deposits in Age-Related Macular Degeneration †

**DOI:** 10.3390/jcm13144131

**Published:** 2024-07-15

**Authors:** Marlene Saßmannshausen, Leyla Sautbaeva, Leon Alexander von der Emde, Marc Vaisband, Kenneth R. Sloan, Jan Hasenauer, Frank G. Holz, Thomas Ach

**Affiliations:** 1 Department of Ophthalmology, University Hospital Bonn, Venusberg-Campus 1, 53127 Bonn, Germanyleon.von_der_emde@ukbonn.de (L.A.v.d.E.);; 2 Life & Medical Sciences Institute, University of Bonn, 53115 Bonn, Germany; 3 Department of Internal Medicine III with Haematology, Medical Oncology, Haemostaseology, Infectiology and Rheumatology, Oncologic Center, Paracelsus Medical University, 5020 Salzburg, Austria; 4 Salzburg Cancer Research Institute—Laboratory for Immunological and Molecular Cancer Research (SCRI-LIMCR), Cancer Cluster Salzburg, 5020 Salzburg, Austria; 5 Department of Computer Science, University of Alabama at Birmingham, Birmingham, AL 35294, USA; 6 Helmholtz Center Munich—German Research Center for Environmental Health, Institute of Computational Biology, 85764 Neuherberg, Germany

**Keywords:** retro mode, subretinal drusenoid deposits, drusen, age-related macular degeneration, imaging

## Abstract

**Background:** Drusen and drusenoid deposits are a hallmark of age-related macular degeneration (AMD). Nowadays, a multimodal retinal imaging approach enables the detection of these deposits. However, quantitative data on subretinal drusenoid deposits (SDDs) are still missing. Here, we compare the capability of en-face drusen and SDD area detection in eyes with non-exudative AMD using conventional imaging modalities versus Retro mode imaging. We also quantitatively assess the topographic distribution of drusen and SDDs. **Methods:** In total, 120 eyes of 90 subjects (mean age ± standard deviation = 74.6 ± 8.6 years) were included. Coherent en-face drusen and SDD areas were measured via near-infrared reflectance, green (G-) and blue (B-) fundus autofluorescence (AF), and Retro mode imaging. Drusen phenotypes were classified by correlating en-face drusen areas using structural high-resolution spectral domain optical coherence tomography. The topographic distribution of drusen was analyzed according to a modified ETDRS (Early Treatment of Diabetic Retinopathy Study) grid. Intraclass correlation coefficient (ICC) analysis was applied to determine the inter-reader agreement in the SDD en-face area assessment. **Results:** The largest coherent en-face drusen area was found using Retro mode imaging with a mean area of 105.2 ± 45.9 mm^2^ (deviated left mode (DL)) and 105.4 ± 45.5 mm^2^ (deviated right mode (DR)). The smallest en-face drusen areas were determined by GAF (50.9 ± 42.6 mm^2^) and BAF imaging (49.1 ± 42.9 mm^2^) (*p* < 0.001). The inter-reader agreement for SDD en-face areas ranged from 0.93 (DR) to 0.70 (BAF). The topographic analysis revealed the highest number of SDDs in the superior peripheral retina, whereas sub-retinal pigment epithelium drusen were mostly found in the perifoveal retina. Retro mode imaging further enabled the detection of the earliest SDD stages. **Conclusions:** Retro mode imaging allows for a detailed detection of drusen phenotypes. While hundreds/thousands of SDDs can be present in one eye, the impact of SDD number or volume on AMD progression still needs to be evaluated. However, this new imaging modality can add important knowledge on drusen development and the pathophysiology of AMD.

## 1. Introduction

Age-related macular degeneration (AMD) is a progressive disease affecting the outer retina of the macula, and is a major cause of irreversible vision loss in the aging population worldwide [1]. Clinically, early and intermediate AMD stages are characterized by the presence of drusen and/or pigmentary changes in the retinal pigment epithelium (RPE), as seen on color fundus photography [2]. Recent developments, including spectral domain optical coherence tomography (SD-OCT) and fundus autofluorescence (FAF), have led to the description of new AMD-related biomarkers.

Conventional drusen consist of extracellular material located between the basal lamina of the RPE and the inner collagenous layer of Bruch’s membrane (BM), and have therefore been termed as sub-RPE drusen [3,4,5]. In contrast, subretinal drusenoid deposits (SDDs) have been defined as “a yellow interlacing network” and are located in the subretinal space [6,7,8,9,10]. In recent years, clinical imaging studies have revealed SDDs as a high-risk factor for AMD progression in both neovascular and geographic atrophy (GA) [10,11,12,13,14].

At the same time, the detailed pathophysiology behind the formation of SDDs, however, is currently not yet fully understood. Both drusen types, SDDs and sub-RPE drusen, share components such as complement factor H, vitronectin and apolipoprotein E [15]. Unlike sub-RPE drusen, SDDs mainly consist of unesterified cholesterol and lack components of photoreceptors and glial cells, which suggests a different pathologic pathway [15]. SDDs have been detected in multimodal imaging settings, including near-infrared reflectance (NIR), blue and green (wavelength) fundus autofluorescence (FAF) and SD-OCT [16], but SD-OCT imaging has been proposed as the most reliable method to detect SDDs. On SD-OCT scans, SDDs appear as a hyperreflective material in the subretinal space in a dot- or ribbon-like pattern [16,17].

Recently, Retro mode imaging has attracted attention as an innovative and non-invasive imaging modality that uses near-infrared light (790 nm) to capture monochromatic images [18]. In contrast to confocal imaging modalities, Retro mode imaging blocks light from the focused planes, but records only backscattered light from laterally shifted apertures (deviated right (DR) or deviated left (DL)), resulting in a pseudo-three-dimensional en-face view of the fundus [19].

Different groups have reported the use of Retro mode imaging in several retinal diseases, showing its possible additional benefit in a multimodal imaging setting [20,21,22]. Furthermore, with the use of Retro mode imaging, recent studies have described an even higher sensitivity and specificity for the detection of smaller drusen, drusen-like deposits, and SDDs [17,19,21,23].

While sub-RPE drusen volume is a reported risk factor for AMD progression [24,25], it is unclear whether the number or volume of SDDs also correlates with the risk of progression [26]. Therefore, the purpose of this study was to compare the detection capability of SDDs and sub-RPE drusen in Retro mode imaging to established conventional en-face imaging techniques (IR, FAF, multicolor). Furthermore, we quantitatively analyzed the number and topographic distribution of sub-RPE drusen and SDDs across the fundus in a large number of AMD patients based on Retro mode methods. This study will add further knowledge on the significance of drusen phenotypes in the course of AMD.

## 2. Methods

### 2.1. Subjects

This cross-sectional study was conducted at the Department of Ophthalmology, University Hospital Bonn, Germany, between May 2021 and August 2022, and was approved by the University of Bonn Ethics Committee (#125/20, 7 October 2020). All study procedures were conducted in accordance with the tenets of the Declaration of Helsinki. Informed consent was obtained from all study participants before conducting any study procedures, and after detailed explanation of the study’s purpose, procedures and potential consequences.

**Figure 1:**
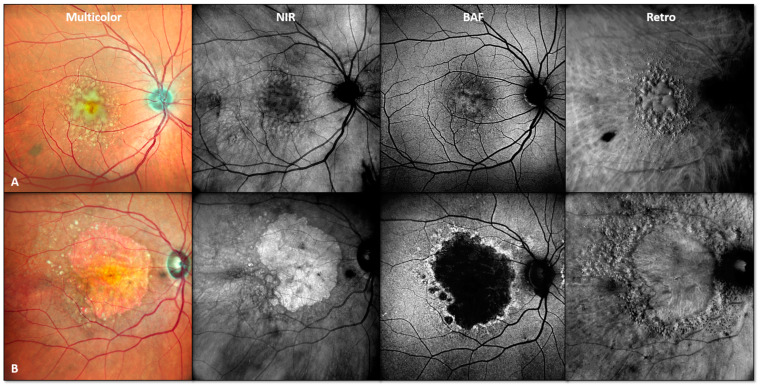
Representative patient examples of study inclusion. Multimodal retinal imaging example of two included study patients with presence of sub-RPE drusen in intermediate AMD ((**A**), male patient, 65 years) and SDDs in non-exudative atrophic ((**B**), female patient, 79 years) AMD. Abbreviations: near-infrared (NIR), blue light fundus autofluorescence (BAF), Retro mode imaging (Retro).

For study inclusion, participants were required to present with either intermediate or non-exudative atrophic AMD according to Ferris et al. [2], as confirmed by multimodal retinal imaging, including NIR, FAF and SD-OCT imaging, in either eye. Figure 1 shows two examples of included study subjects with the presence of sub-RPE drusen in intermediate AMD, and SDDs in non-exudative atrophic AMD. Eyes with significant lens opacity (i.e., cataracts) affecting image quality, any prior history of intraocular surgery (except for cataract surgery), any signs of current or prior active macular neovascularization (MNV) or other retinal diseases, or previous recorded treatment for neovascular AMD were excluded. If both eyes met the inclusion criteria, both eyes were included.

### 2.2. Retinal Imaging Protocol

After pupil dilatation (0.5% tropicamide, 2.5% phenylephrine), participants underwent a detailed and standardized retinal imaging protocol using a Mirante SLO/OCT device (NIDEK Co., Ltd., Japan, 40° × 30°, 1024 × 1024 pixels) with near-infrared reflectance (NIR, 790 nm), green (GAF, 532 nm) and blue fundus autofluorescence (BAF, 488 nm), and Retro mode illumination deviated right (DR) and deviated left (DL) [27]. In addition, high-speed combined and simultaneous confocal scanning laser ophthalmoscopy (cSLO) and SD-OCT (30° × 25°; 121 B-scans centered on the fovea; distance of B-scans—60 µm; averaging automatic real time—25 frames) was performed using an investigational high-resolution SD-OCT device (High-Res OCT, Spectralis, Heidelberg Engineering, Heidelberg, Germany). If not specified further, “drusen” in this study refers to both sub-RPE drusen and SDDs.

### 2.3. Multimodal Retinal Imaging Analysis of the En-Face Drusen Area

**Figure 2:**
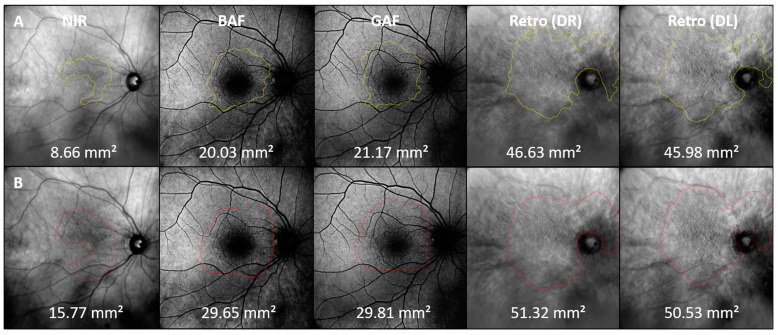
SDD en-face area detection by readers. Example of SDD en-face areas manually outlined by two independent readers (**A**,**B**). NIR: near-infrared; BAF: blue fundus autofluorescence; GAF: green fundus autofluorescence; DR: Retro mode imaging deviated right; DL: deviated left.

Coherent en-face drusen areas were manually labelled and measured in all available en-face imaging modalities (NIR, BAF, GAF, Retro mode DR and DL, considering the corresponding image scaling factor for each image modality) using the freehand tool in the open-source platform Fiji, Version: 2.9.0 (U.S. National Institutes of Health). Retinal areas of geographic atrophy (GA) as well as the optic disc were excluded from the en-face area analysis. Also, for assessing the inter-reader agreement of SDD detection in the en-face areas in multimodal retinal imaging, coherent SDD en-face areas were annotated and measured by two independent readers in a subset of images (example Figure 2). After the consideration of multimodal retinal imaging data for the detection of participants with the sole SDD drusen phenotype, inter-reader analysis for en-face area detection was performed (*n* = 28).

### 2.4. Determination of Topographical Distribution of Sub-RPE and SDD Drusen

For analyzing the topographic distribution of drusen phenotypes, single lesions of sub-RPE and SDD drusen were quantified in a subgroup of 30 eyes of 25 participants. These eyes had the highest image quality across all modalities that were used. Detailed characteristics of this subgroup are presented in Table 1.

First, NIR (which is in parallel with SD-OCT imaging) and Retro mode images were aligned according to vessel bifurcations using a self-customized Fiji, ImageJ plugin (plugin: Register OCT 2). This enabled us to transform the location of lesions detected in the cross-sectional SD-OCT into the en-face Retro mode image (plugin: en-face Lesions OCT). Further, drusen were classified into phenotypes according to their appearance on SD-OCT imaging. In Retro mode imaging, sub-RPE drusen appeared as large, flat, more irregular lesions, and were concentrated at or close to the fovea, while SDDs presented as small, dot- or ribbon-like lesions primarily located in the peripheral retina. Lesions not visible in SD-OCT but visible in Retro mode imaging were categorized according to their characteristic appearance in Retro mode imaging. Hard drusen and cuticular drusen visible in OCT were excluded from the analysis. All customized FIJI plugins are available at https://sites.imagej.net/CreativeComputation/ (accessed on 7 July 2024).

A modified Early Treatment Diabetes Retinopathy Study (mETDRS) grid, centered on the fovea and superimposed on the Retro mode images, was used to quantify drusen distribution for each subfield of the mETDRS grid (Supplementary Figure S1).

### 2.5. Statistical Analysis

Statistical analysis was performed using R (R core Team, ver. 4.2.1). For the SDD en-face areas, inter-reader agreement was determined between two readers with the intra-class correlation coefficient (ICC) and validated as fair (0.21–0.40), moderate (0.41–0.60), substantial (0.61–0.80), or almost perfect (0.81–1.00) according to the categorization proposed by Landis and Koch [28].

*p*-values were corrected for multiple testing using the Bonferroni method. The significance threshold was chosen to be 0.05.

## 3. Results

### 3.1. Patient Demographics

One hundred and twenty eyes of ninety patients with intermediate- and late-stage non-neovascular AMD (mean age ± standard deviation (SD): 74.6 ± 8.6 years; median: 74 years; 0.25–0.75 interquartile range: 68.8–81 years; 81 females; 50 pseudophakic eyes) were included in this study. The mean best-corrected visual acuity (BCVA) was 0.2 ± 0.3 logMAR (Snellen equivalent 20/32 ± 20/40). Detailed cohort characteristics are presented in Table 1.

### 3.2. Quantification of En-Face Drusen Areas

**Figure 3:**
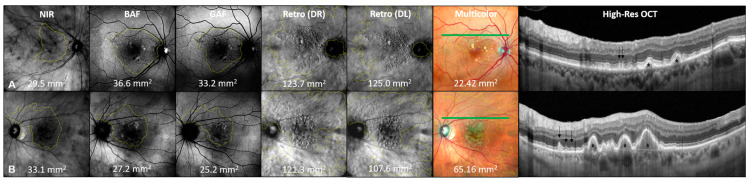
Quantification of en-face drusen area in multimodal imaging. Multimodal retinal imaging example of en-face drusen quantification in two representative female patients with predominant presence of sub-RPE drusen (patient (**A**), 74 years) and SDDs (patient (**B**), 77 years) with intermediate AMD. En-face area values are given in mm^2^ per imaging modality and patient. Notice the significantly greater en-face areas detected by Retro (DR) and Retro (DL) imaging in both cases. NIR: near-infrared; BAF: blue fundus autofluorescence; GAF: green fundus autofluorescence; DR: deviated right Retro mode imaging: DL: deviated left Retro mode imaging; multicolor corresponds to High-Res OCT B-scan. The position of the High-Res OCT B-scan is highlighted by the green line on the multicolor image. In the High-Res OCT B-scan, the presence of SDDs (black arrows) and sub-RPE drusen (black stars) are highlighted.

The largest en-face area covered by drusen was determined in the Retro mode, with a mean area of 105.2 ± 45.9 mm^2^ (DL) and 105.4 ± 45.5 mm^2^ (DR). Compared to Retro mode imaging, the overall mean en-face areas assessed in the conventional retinal imaging modalities were smaller, with 53.2 ± 44.7 mm^2^ in NIR, 50.9 ± 42.6 mm^2^ in GAF, 49.1 ± 42.9 mm^2^ in BAF, and 60.6 ± 45.5 mm^2^ in multicolor imaging. In non-parametric testing, the difference was statistically highly significant (*p* < 0.001) for all conventional retinal imaging modalities: NIR, GAF, BAF and multicolor imaging. Two representative examples of en-face drusen area quantification in study participants with the presence of sub-RPE drusen and SDDs are given in Figure 3.

### 3.3. Inter-Reader Agreement for SDD Area Determination in the En-Face Mode

When comparing the inter-reader agreement for SDD en-face area determination (*n* = 28), the repeatability of measurements varied between readers, with the highest consensus found for the DR (ICC = 0.930; confidence interval: 0.860 to 0.979) and DL (0.936; 0.870 to 0.970) Retro mode imaging modalities, followed by NIR imaging (0.822; 0.660 to 0.910). Moderate-to-substantial agreement between readers was found for the en-face SDD determination in the GAF (0.737; 0.510 to 0.870) and BAF (0.696; 0.440 to 0.580) en-face imaging methods. A patient example for inter-reader agreement in the en-face area determination is given in Figure 2.

### 3.4. Topographical Distribution of Drusen Phenotypes Using a Modified ETDRS (mETDRS) Grid

**Figure 4:**
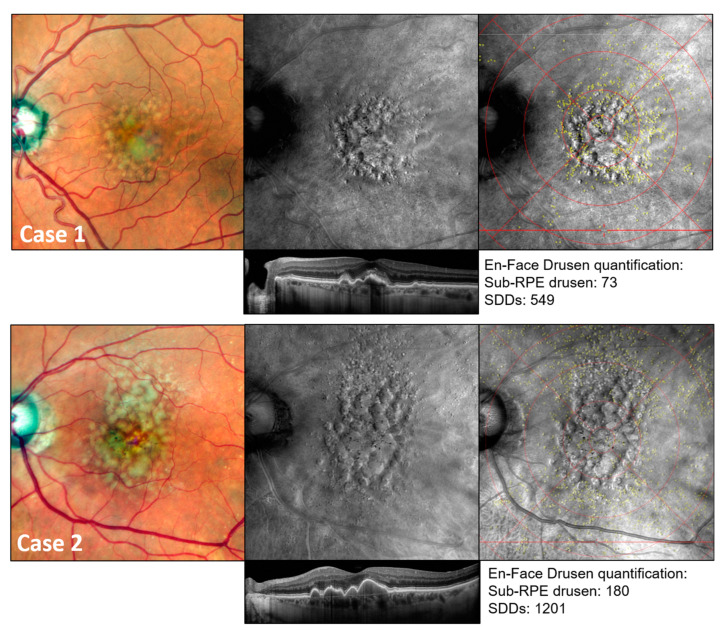
Multimodal retinal imaging example of sub-RPE drusen and SDD quantification using modified ETRDS grid. Multimodal retinal imaging example of drusen number quantification in two representative female patients aged 70 (case 1) and 77 (case 2) with intermediate AMD. Visible lesions were located in Retro mode as well as in OCT imaging and categorized into drusen phenotypes according to their typical structural alterations as seen in OCT imaging. Overlaying a modified ETDRS grid (details presented in Supplementary Figure S1) allowed the topographic analysis of SDDs (yellow dots) and sub-RPE drusen (slight white rings).

**Figure 5:**
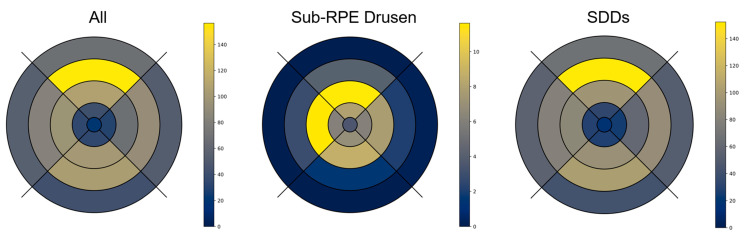
Topographical distribution of sub-RPE drusen and SDD according to modified ETDRS grid. This figure shows a graphical illustration of the topographical distribution of all drusen, as well as different drusen phenotypes in Retro mode imaging within the study subcohort according to a modified ETDRS grid. The results are presented as mean number of drusen per ETDRS subfield. Unit: number of lesions/mm^2^.

This sub-analysis was performed on 30 eyes of 25 participants (72.2 ± 5.7 years; median: 72 years; interquartile range 0.25–0.75: 69–77 years; 21 females; 11 pseudophakic eyes). Two representative patient examples of multimodal en-face drusen quantification are shown in Figure 4. Using Retro mode imaging, the largest numbers of sub-RPE drusen were detected at the fovea and perifovea. In contrast, the highest numbers of SDDs were detected in the superior peripheral subfields (segment 15) of the mEDTRS grid (mean number of SDDs: 152.33 ± 148.09), while sub-RPE drusen were mainly found in segments 11 (11.63 ± 21.00) and 12 (11.57 ± 18.13). Detailed results on the topographical distribution of drusen phenotypes according to the ETDRS subfields given in number of lesions/mm^2^, as well as the total number of drusen per ETDRS subfield are presented in Figure 5 and Supplementary Table S1.

### 3.5. Retro Mode Detects Early Stages of SDDs

**Figure 6:**
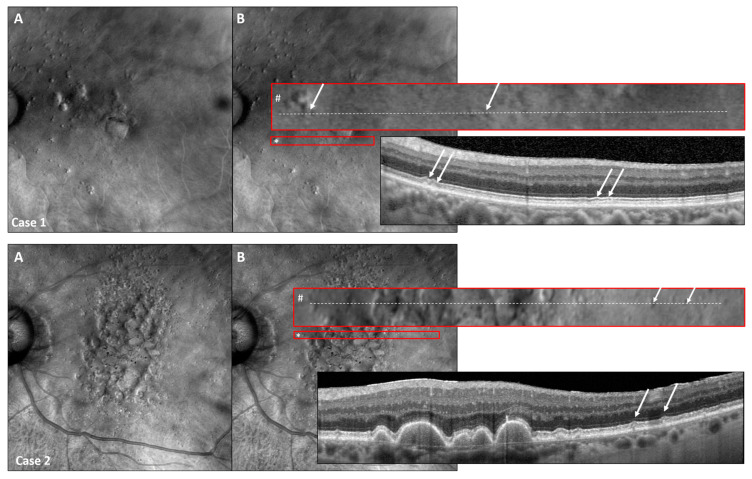
Retro mode detects early SDD stages. Two representative study eyes presenting with early SDD stages detectable with Retro mode and high-resolution SD-OCT imaging (High-Res OCT). Consider the Retro mode image (**A**) with the red encircling the original size of the displayed area (*) as well as the enlarged red-encircled (#) area of interest shown in image (**B**). The exact location of the corresponding OCT scan is shown as a white-dashed line in the enlarged red rectangle (#). As SDDs were detectable in both patient examples, Retro mode imaging is capable of detecting even slight subretinal alterations representing the early stages of SDD (white arrows) [7].

Using the Retro mode, we were able to detect even the early stages of SDDs (stage 1, as defined by Zweifel et al. [7]), with SDD stages confirmed in SD-OCT imaging (Figure 6).

## 4. Discussion

In this study, we used Retro mode imaging to quantify sub-RPE drusen and SDD numbers and to analyze the distribution in intermediate- and atrophic late-stage AMD patients, and validated this method against other fundus imaging techniques. Retro mode imaging enabled the clear detection of SDDs. In addition, by linking cross-sectional SD-OCT and en-face Retro mode imaging, the individual drusen types could be annotated and localized at the posterior pole, showing characteristic distribution across the fundus for both drusen phenotypes.

Alongside sub-RPE drusen and pigmentary abnormalities, SDDs have been recognized as the third major risk factor for progression to late AMD, in particular GA [13]. The exact pathophysiology of SDDs is still unclear, but their formation may be caused by rod photoreceptor dysfunctions [29,30]. Other theories suggest associations with choroidal thinning and reduced blood flow, as confirmed by OCTA [31].

The number of detectable SDDs exceeds the number of sub-RPE drusen by far, and SDDs are found numerously, but not exclusively, in the outer ETDRS rings, in particular the superior peripheral retina. These findings mostly coincide with previous studies exploring the distribution of SDDs [29], suggesting that SDD distribution follows the abundancy of rod photoreceptors, with the highest density of rods outside the vessel arcades [30,32,33]. In contrast to our study findings, SDDs have not been reported near the fovea so far, possibly because of the lack of more sensitive imaging modalities, such as OCT and Retro mode illumination [34,35].

Various studies have assessed the prevalence of SDDs in patients with AMD. The Melbourne Collaborative Cohort Study reported a prevalence of 1.8% in eyes with intermediate AMD and 12.3% in eyes with late AMD based on color fundus photography [36]. The numbers increased significantly by including cross-sectional high-resolution SD-OCT in imaging protocols, with 38.4% of right and 35.8% of left eyes being affected by any form of AMD showing SDDs in the SD-OCT [37]. In our study, all eyes included for the sub-analysis presented SDDs in Retro mode imaging. The high prevalence of SDDs in patients with AMD could imply that SDD presence is significant in disease pathogenesis and progression. However, a high prevalence in this elderly cohort could also mean that SDDs are, to some extent, a sign of the aging retina—similar to druplets (sub-RPE drusen < 63 µm), which counted towards AMD disease severity in the AREDS classification [38], but which were later considered a phenomenon of the aging eye, as defined in the Beckman classification [2].

In contrast to confocal systems, Retro mode imaging uses backscattered light captured by a detection camera, while light from the focused planes is blocked. Retro mode imaging generates pseudo three-dimensional images, which then enable the discrimination of irregularities in the outer retina and adjacent tissue. Therefore, in recent years, Retro mode imaging has been used to characterize various retinal pathologies, including AMD [36,39,40]. Since both sub-RPE drusen and SDDs present as piled-up material between corresponding layers and negatively affect the regular architecture of the retina, these (and other) lesions appear as pseudo three-dimensional elevations in Retro mode imaging [17,27,28,29,30].

Previously, conventional en-face imaging modalities have been used to confirm the presence of SDDs in AMD-affected eyes, including NIR, fundus autofluorescence and color fundus photography. In this study, Retro mode imaging proved to be the most sensitive en-face method for the detection of SDDs. The above-mentioned pseudo three-dimensional imaging might be superior to imaging techniques that plot only two-dimensional data. In the future, further developments like en-face SD-OCT might be able to create similar drusen and SDD distribution maps, as recently demonstrated [14,41]. However, a direct comparison between Retro mode and en-face OCT is still missing. The advantage of imaging in Retro mode is that no time-consuming image post-processing is necessary; in particular, no segmentation of the retinal layers is needed, as in SD-OCT.

While sub-RPE drusen volume has already been acknowledged as a risk factor for AMD progression, there are still no widely accepted quantitative parameters that can be used to determine SDDs’ impact on AMD progression [24,25,42]. Imaging methods that enable the fast and accurate quantification (number, distribution, area of occupation) of SDDs are, therefore, desirable. While annotation was undertaken manually in our study, future artificial-intelligence-assisted automatic algorithms might help to speed up this process in a larger study cohort and in long-term follow-up.

Interestingly, Retro mode imaging was able to detect subtle drusen-like formations, suggesting that the earliest stages of SDDs (SDD type 1) could be detected [37]. This gives hope for future studies investigating the development and clinical impact of SDDs, as the earliest changes can be identified and functional examinations can be carried out in direct function–structure correlations at the affected sites [43,44]. Furthermore, better insights into the early stages of AMD might also help to increase our knowledge on SDD lesion development, progression, and regression, as previously shown for sub-RPE drusen [45].

Our study has its limitations. Firstly, confluent sub-RPE drusen hindered manual drusen quantification, thus limiting the number of suitable study eyes for our sub-analysis. Peripheral SDDs (in some cases outside of the available SD-OCT scans) were diagnosed according to their characteristic appearance in Retro mode imaging, therefore risking the incorrect classification of drusen sub-types. Using structural OCT images, we were able to classify SDDs into different stages. However, in future studies, and to prevent over-sensitive detection, a multimodal retinal imaging approach for SDD detection should validate if the use of Retro mode enables for a better differentiation of SDD stages compared to conventional imaging modalities. Additionally, discrepancies resulting from imperfect point-to-point correlation between NIR imaging and SD-OCT, as well as the manual alignment of Retro mode imaging and OCT, could have complicated the correct categorization of drusen. An automatic approach may resolve this challenge in the future.

However, this is the first study to quantify the number of visible SDDs in Retro mode imaging and simultaneously confirm the correct identification via OCT, while excluding retinal areas unaffected by lesions typical for AMD.

In conclusion, Retro mode imaging proved to be the most sensitive approach to determine the presence of even the smallest SDDs among the en-face imaging modalities. Therefore, we suggest including this novel imaging modality in the established multimodal imaging setting used to diagnose SDDs. This will add further knowledge on their significance in eyes affected by AMD.

## Figures and Tables

**Table 1:** Study cohort characteristics.

**Participants**	**Study Cohort** **En-Face Drusen Area Quantification**	**Study Cohort** **Single Drusen Quantification**
Number of study eyes	120	30
Number of patients	90	25
Mean age (years ± SD) [range]	74.6 ± 8.6 [55–91]	72.2 ± 5.7 [59–85]
Gender (male)	39	9
BCVA [logMAR] (±SD)	0.2 ± 0.3	0.2 ± 0.2
Pseudophakic	50	11

BCVA: best-corrected visual acuity; SD: standard deviation.

## Data Availability

Data are available upon reasonable request.

## Supplementary Materials

The following supporting information can be downloaded at: https://www.mdpi.com/article/10.3390/jcm13144131/s1, Figure S1: Modified ETDRS grid. Table S1: Number of sub-RPE drusen and SDDs in the subfields of the modified ETDRS (mETDRS) grid.
