# Supplementary material for: Retro Mode Imaging for Detection and Quantification of Sub-RPE Drusen and Subretinal Drusenoid Deposits in Age-Related Macular Degeneration
Source: J Clin Med. 2024 Jul 15;13(14):4131. doi: 10.3390/jcm13144131 (PMC11278487; doi:10.3390/jcm13144131)
Supplement: Supplementary file 1 [file jcm-13-04131-s001.zip › jcm-3066331-supplementary.pdf]

Supplemental Material:

Supplemental Figure S1: Modified ETDRS grid.

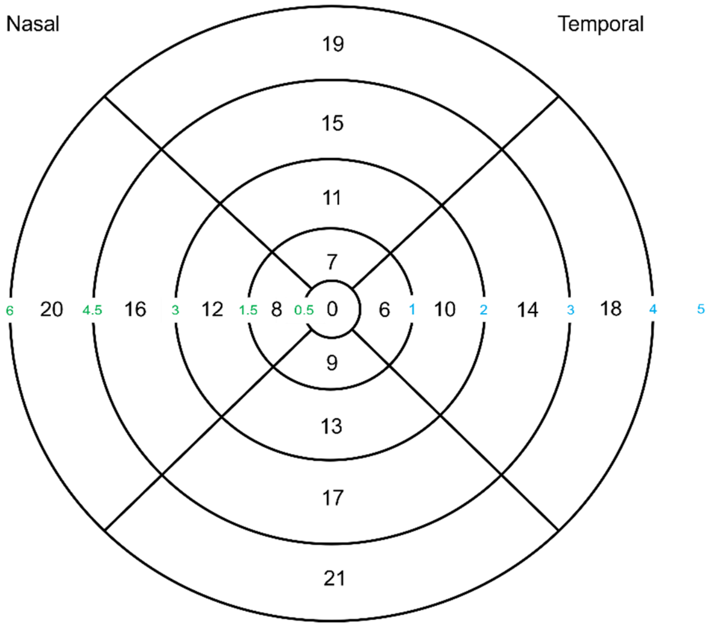

A modified ETDRS (mETDRS) grid was applied for the topographical analysis of drusen distribution across the posterior pole. The mETDRS grid consists of the rings 1 to 5 (blue), with ring 5 including all drusen located outside of rings 1 to 4.

Each ring is further divided into four subfields. The green numbers show the radial distance from the foveal center of the ETDRS grid (in mm).

The temporal retina is represented by segments 6, 10, 14 and 18, the nasal retina by segments 8, 12, 16 and 20.

Supplemental Table S1: Total number of sub-RPE drusen and SDDs in the subfields of the modified ETDRS (mETDRS) grid.

mETDRS subfields	All (Mean ± SD)	Number of sub-RPE Drusen (Mean ± SD)	Number of SDDs (Mean ± SD)
**0**	20.2 ± 22.6	3.6 ± 3.8	16.6 ± 22.5
**1**	136.6 ± 100.2	26.2 ± 20.7	110.2 ± 100.2
**2**	366.6 ± 258.7	38.9 ± 47.6	327.5 ± 250.7
**3**	430.1 ± 442.0	10.5 ± 16.9	419.6 ± 437.3
**4**	212.3 ± 251.8	0.5 ± 0.9	211.8 ± 251.2
**5**	10.5 ± 21.2	0.0 ± 0.0	10.5 ± 21.2
**6**	30.8 ± 23.8	6.1 ± 6.9	24.6 ± 22.2
**7**	41.2 ± 31.0	8.0 ± 7.6	33.1 ± 30.7
**8**	34.8 ± 28.0	6.1 ± 5.5	28.6 ± 28.5
**9**	34.7 ± 27.8	6.9 ± 6.5	27.7 ± 27.5
**10**	68.4 ± 59.0	7.8 ± 9.4	60.6 ± 54.9
**11**	107.1 ± 84.9	11.6 ± 21.0	95.5 ± 82.5
**12**	96.3 ± 82.1	11.6 ± 18.1	84.7 ± 78.4
**13**	100.8 ± 67.2	8.7 ± 11.0	92.1 ± 65.3
**14**	91.2 ± 118.0	2.1 ± 6.8	89.2 ± 115.7
**15**	156.5 ± 149.5	4.1 ± 6.9	152.3 ± 148.1
**16**	82.7 ± 90.2	2.9 ± 4.7	79.8 ± 88.7

**17**	104.1 ± 125.4	1.6 ± 4.9	102.5 ± 124.6
**18**	52.2 ± 80.3	0.3 ± 0.8	52.0 ± 79.7
**19**	67.7 ± 76.1	0.1 ± 0.2	67.6 ± 76.1
**20**	55.0 ± 62.0	0.1 ± 0.3	54.8 ± 62.1
**21**	42.2 ± 63.0	0.1 ± 0.3	42.1 ± 63.0
**Total mETDRS grid**	1136.3 ± 835.2	76.9 ± 66.6	1059.1 ± 829.1
